# Supplementary material for: A JAK/STAT-Pdk1-S6K axis bypasses systemic growth restrictions to promote regeneration
Source: Nat Commun. 2025 Dec 6;16:10944. doi: 10.1038/s41467-025-66995-z (PMC12686489; doi:10.1038/s41467-025-66995-z)
Supplement: Supplementary file 1 — Supplementary Information [file 41467_2025_66995_MOESM1_ESM.pdf]

A JAK/STAT-Pdk1-S6K axis bypasses systemic growth restrictions to promote regeneration

Ananthakrishnan Vijayakumar Maya [1,2,3], Lena Neuhaus [4,5,6], Liyne Nogay [1,2,3], Aakriti Singh [1,2,3], Lara Heckmann [1, 2, 3], Isabelle Grass [1, 2], Jörg Büscher [7], Katrin Kierdorf [5,6,8], Anne-Kathrin Classen [1, 2, 8] *

* Corresponding author: anne.classen@biologie.uni-freiburg.de

Supplementary Figures S1-S8

Supplementary Tables S1, S2

Supplementary File S1

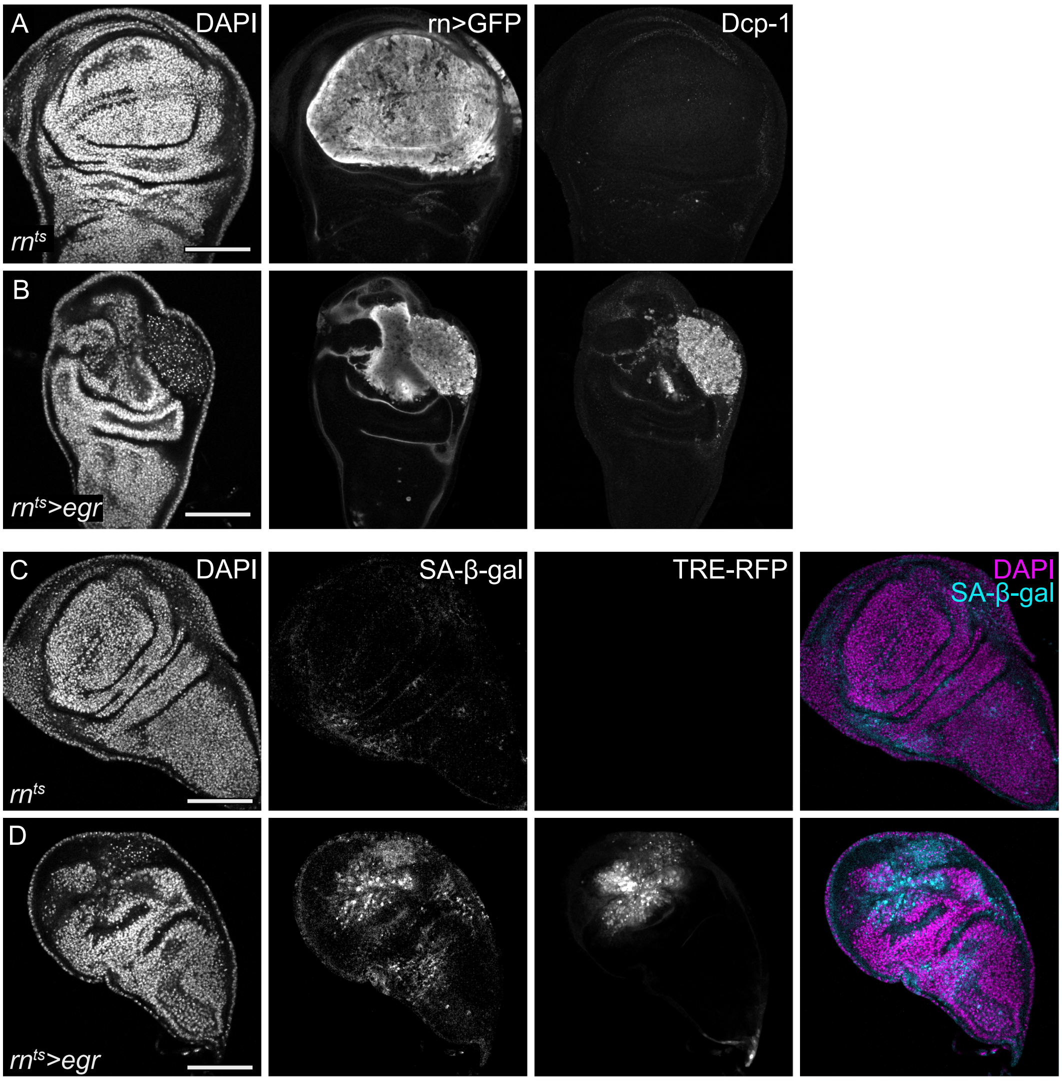
A
DAPI
rn>GFP
Dcp-1
rn^ts
B
rn^ts>egr
C
DAPI
SA-β-gal
TRE-RFP
DAPI
SA-β-gal
rn^ts
D
rn^ts>egr

S1

Figure S1. Cell cycle adaptations in response to inflammatory tissue damage

A, B. Control wing disc (A) and a wing discs (B) after 24 hours of *egr*-expression within the wing pouch domain under the control of the *rn*-GAL4 driver (*rn*^{ts} and *rn*^{ts}>*egr*, respectively). A co-expressed UAS-GFP construct visualizes the *rn*-GAL4 expression domain. Staining for cleaved Dcp-1 visualizes apoptosis. Discs were stained with DAPI to visualize nuclei. Please note the GFP-expression domain in (B) which does not stain for cDcp-1 and represents surviving cells with high levels of JNK activity. Levels of Dcp-1 were previously quantified (La Fortezza, Schenk et al. 2016, Cosolo, Jaiswal et al. 2019, Jaiswal, Egert et al. 2023).

C, D. Analysis of senescence-associated β-galactosidase (SA-β-gal) activity (cyan or grey) in control (C) and *egr*-expressing wing disc (D). TRE-RFP visualizes JNK-pathway activity. Discs were stained with DAPI to visualize nuclei (magenta or grey). SA-β-gal activity was already previously reported and quantified (Floc'hlay, Balaji et al. 2023, Jaiswal, Egert et al. 2023).

Scale bars: 100 µm.

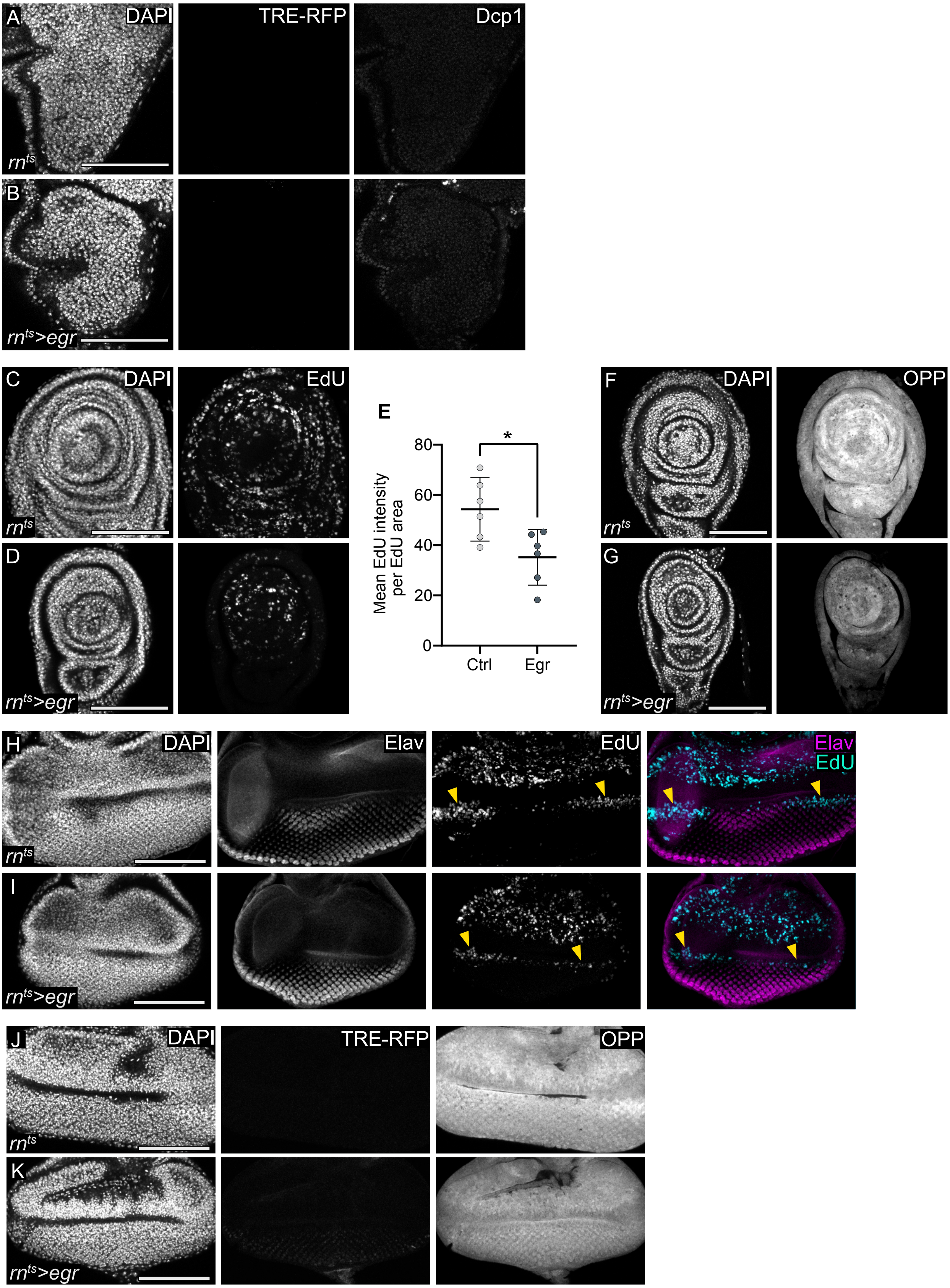
A
DAPI
TRE-RFP
Dcp1
rnts
B
rnts>egr
C
DAPI
EdU
rnts
D
rnts>egr
E
*
Mean EdU intensity per EdU area
80
60
40
20
0
Ctrl
Egr
F
DAPI
OPP
rnts
G
rnts>egr
H
DAPI
Elav
EdU
Elav
EdU
rnts
I
rnts>egr
J
DAPI
TRE-RFP
OPP
rnts
K
rnts>egr

S2

Figure S2. Metabolic adaptation in peripheral tissues

A, B. Nota of control (A) and wing disc expressing *egr* in the pouch (B). TRE-RFP visualizes JNK-pathway activity and staining for cleaved Dcp-1 visualizes apoptosis. Discs were stained with DAPI to visualize nuclei.

C, D. EdU incorporation to visualize DNA replication in leg imaginal discs, dissected from larvae containing either control wing disc (C) or *egr*-expressing wing discs (D). Discs were stained with DAPI to visualize nuclei.

E. Mean EdU intensity per EdU-positive area quantified in leg imaginal discs, dissected from larvae containing either control wing disc (C) or *egr*-expressing wing discs (D), serving as a proxy for DNA replication speed. Mean and 95% CI are shown. Statistical significance was tested using an two-tailed Unpaired t-test with a p-value = 0.0154 (control: n=6, *egr*-expressing disc: n=6).

F, G. Protein synthesis visualized by O-proparyl-puromycin (OPP) incorporation into newly synthesized proteins in leg imaginal discs, dissected from larvae containing either control wing disc (F) or *egr*-expressing wing discs (G). Discs were stained with DAPI to visualize nuclei.

H, I. EdU incorporation (cyan or grey) to visualizes DNA replication in eye imaginal discs, dissected from larvae with control (H) or *egr*-expressing (I) wing imaginal discs. Eye disc differentiating photoreceptors are marked with Elav staining (magenta or grey). Sum projection of multiple image slices to visualize the second mitotic wave (SMW) and the yellow arrowheads point to the expected location of the SMW.

J, K. Protein synthesis visualized by OPP incorporation in the eye imaginal disc (same as **Fig 2.O and P**), dissected from larvae with control (J,) or *egr*-expressing (K) wing imaginal discs using *rn*-GAL4 driver. TRE-RFP visualizes JNK-pathway activity and discs were stained with DAPI to visualize nuclei.

Scale bar: 100 µm.

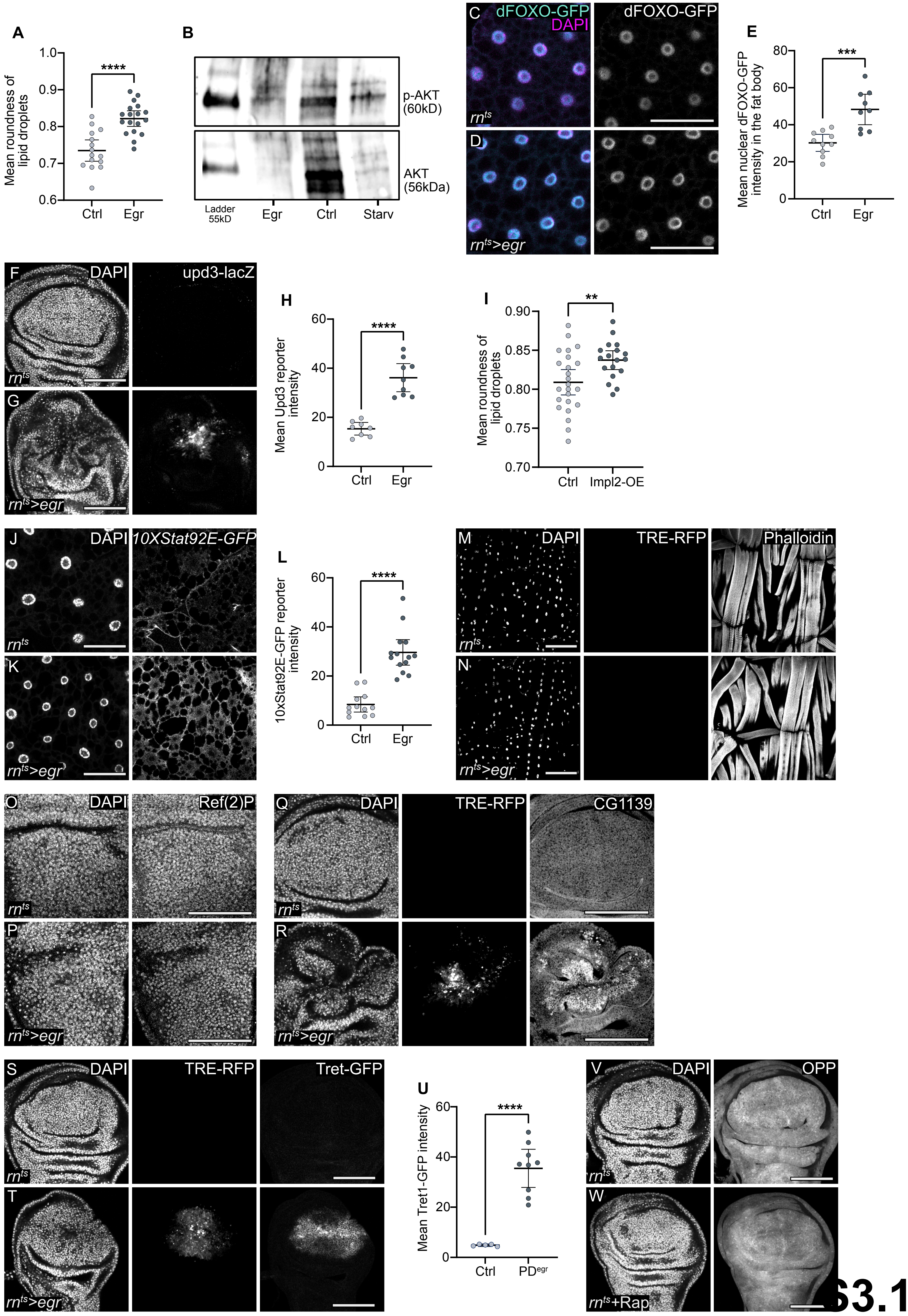
A
Mean roundness of lipid droplets
****
Ctrl
Egr
B
p-AKT (60kD)
AKT (56kDa)
Ladder 55kD
Egr
Ctrl
Starv
C
dFOXO-GFP
DAPI
D
E
Mean nuclear dFOXO-GFP intensity in the fat body
***
F
DAPI
upd3-lacZ
G
H
Mean Upd3 reporter intensity
I
Mean roundness of lipid droplets
**
Impl2-OE
J
10XStat92E-GFP
K
L
10xStat92E-GFP reporter intensity
M
TRE-RFP
Phalloidin
N
O
Ref(2)P
P
Q
CG1139
R
S
Tret-GFP
T
U
Mean Tret1-GFP intensity
PDegr
V
OPP
W
rnts+Rap

S3.1

Figure S3.1 Elevated levels of nutrient transporters and mTORC1 signaling support regenerative proliferation

---

A. Quantification of roundness of lipid droplets in the fat body dissected from larvae with control (**Fig.3A**) or *egr*-expressing (**Fig.3B**) wing imaginal discs. Mean and 95% CI are shown. Roundness is measured as the ratio of the area to the square of the length of the major axis. A perfect circle has a roundness value of 1, while values approaching zero indicate a more elongated shape. Statistical significance of was tested using an two-tailed Unpaired t-test. Mean roundness, p-value < 0.0001. (control: n=15, *egr*-expression in discs: n=18).

B. Protein expression levels of p-AKT (Ser473) and AKT by western blot in the fat body dissected from larvae with control or *egr*-expressing wing imaginal discs or 24 h starved larvae.

C, D. Expression of dFOXO-GFP (cyan or grey) in fat body, dissected from larvae with control (I) or *egr*-expressing (J) wing imaginal discs. Fat bodies were stained with DAPI to visualize nuclei (magenta).

E. Mean nuclear dFOXO-GFP intensity in fat body, dissected from larvae with control or *egr*-expressing wing imaginal discs. Statistical significance was tested using an two-tailed unpaired t-test, p-value = 0.0003 (control: n=10, *egr*-expressing disc: n=9).

F, G. Expression of Upd3-lacZ in control (C) and *egr*-expressing discs (D). Discs were stained with DAPI to visualize nuclei.

H. Upd3-lacZ intensity quantified in the pouch of control wing discs and in the JNK-signaling domain of *egr*-expressing discs. Mean and 95% CI are shown and statistical significance was tested using two-tailed Welch's t-test with p-value < 0.0001 (control: n=8, *egr*-expressing disc: n=9).

I. Quantification of roundness of lipid droplets in the fat body dissected from larvae with either control discs or wing imaginal discs expressing ImpL2 for 24 h under the control of *rn*-GAL4. Roundness is measured as the ratio of the area to the square of the major axis. A perfect circle has a roundness value of 1, while values approaching zero indicate more elongated shape. Statistical significance was tested using the two-tailed Unpaired t-test, p-value=0.0088 (control: n=24, *egr*-expression in disc: n=18).

J, K. Activity of the JAK/STAT reporter *STAT92E-GFP* in the fat body dissected from larvae with control (J) or *egr*-expressing (K) wing imaginal discs. DAPI visualizes nuclei.

L. Quantification of *STAT92E-GFP* reporter intensity in the fat body dissected from larvae with control or *egr*-expressing wing imaginal discs. Mean and 95% CI are shown and statistical significance was tested using the two-tailed Mann-Whitney test, p-value < 0.0001 (control: n=12, *egr*-expression in disc: n=14).

M, N. *Drosophila* larval body wall muscles dissected from larvae containing either control (M) or *egr*-expressing (N) wing discs. Actin was visualized using Phalloidin and TRE-RFP visualizes JNK-pathway activation. Muscles were stained with DAPI to visualize nuclei. Please note that muscles do not activate JNK-signaling in response to inflammatory cues.

O, P. Nota of control (O) and *egr*-expressing wing disc (P) stained for the autophagy marker Ref(2)P. Discs were stained with DAPI to visualize nuclei.

Q, R. Staining for the putative alanine, glycine and proline amino acid transporter Arcus (CG1139) in control (Q) and *egr*-expressing discs (R). TRE-RFP visualizes JNK-pathway activation. Discs were stained with DAPI to visualize nuclei.

S, T. Expression of Tret1.1-GFP in control (S) and *egr*-expressing discs (T). TRE-RFP visualizes JNK-pathway activation. Discs were stained with DAPI to visualize nuclei.

U. Mean Tret1.1-GFP intensity quantified in the pouch of control discs and the proliferative domain of *egr*-expressing discs. Mean and 95% CI are shown. Statistical significance was tested using the two-tailed Welch's t-test, p-value < 0.0001 (control: n=5, *egr*-expressing discs: n=9).

V, W. Protein synthesis visualized by OPP incorporation in control (V) and larvae fed on food with rapamycin (200 µM) (W) for 24 h during the temperature shift.

Scale bar: 100 µm. Fluorescence intensities are reported as arbitrary units.

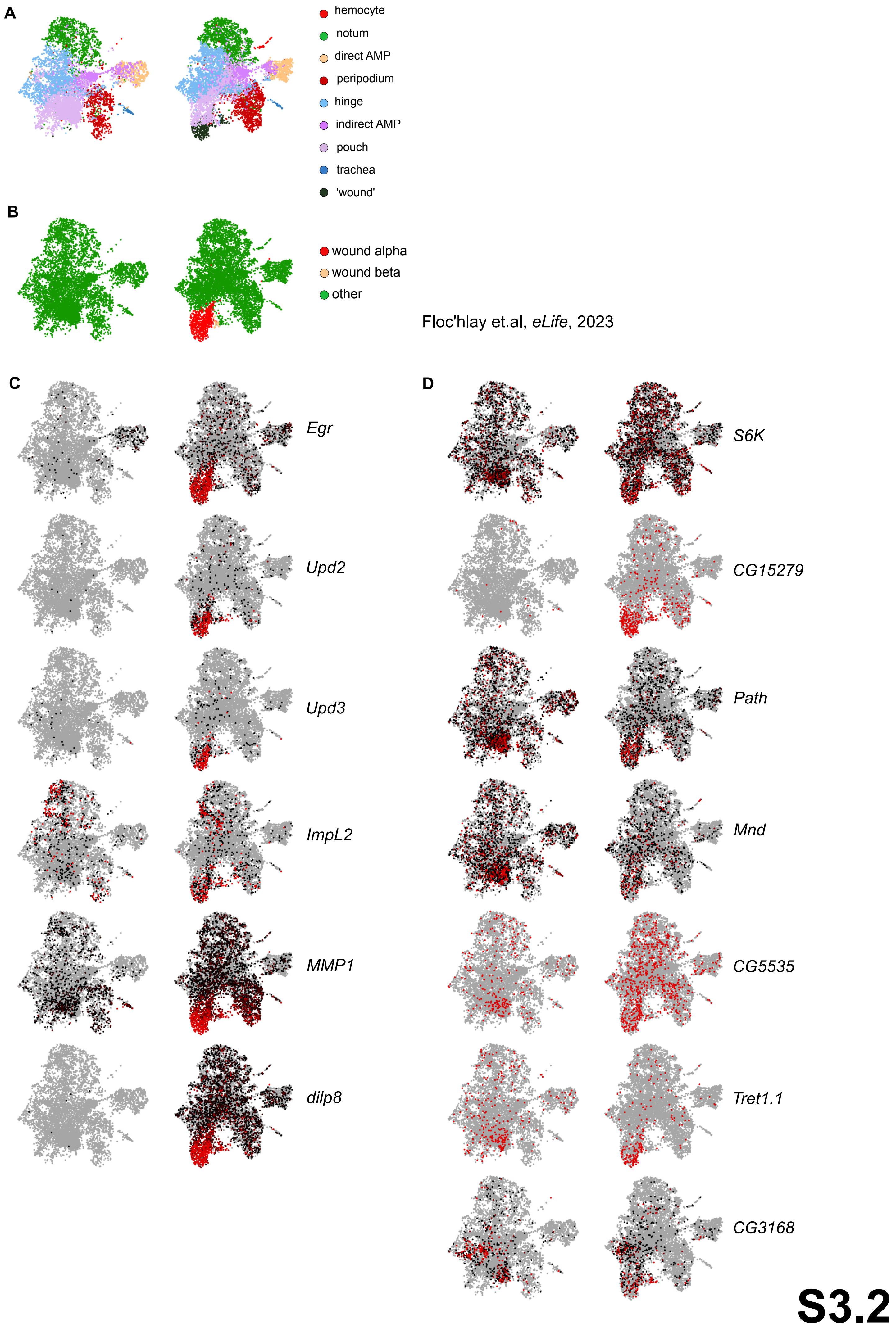

A
hemocyte
notum
direct AMP
peripodium
hinge
indirect AMP
pouch
trachea
'wound'
B
wound alpha
wound beta
other
Floc'hlay et.al, eLife, 2023
C
Egr
Upd2
Upd3
ImpL2
MMP1
dilp8
D
S6K
CG15279
Path
Mnd
CG5535
Tret1.1
CG3168
S3.2

Figure S3.2 Elevated levels of nutrient transporters and mTORC1 signaling support regenerative proliferation

A.B UMAP plots generated using the Scope Wing Atlas created from control and *egr*-expressing discs analysed by single-cell RNA-Seq technology (Floc'hlay, Balaji et al. 2022). Different cell populations are color-coded. The analysis in (Floc'hlay, Balaji et al. 2022) indicating the emergence of a 'wound' cluster in *egr*-expressing discs, subdivided into two clusters with signatures characteristic of senescent cells (beta) and with characteristic of a cell and tissue damage response signature (alpha).

C. ScRNA-Seq UMAP plots showing the elevated transcript levels of secreted paracrine ligands with known interorgan signaling roles. Grey indicates low expression, and red indicates high expression of transcripts.

D. scRNA-Seq UMAP plot displaying significantly elevated transcript levels detected for different nutrient transporters and S6K (Floc'hlay, Balaji et al. 2023). Grey indicates low expression, and red indicates high expression of transcripts.

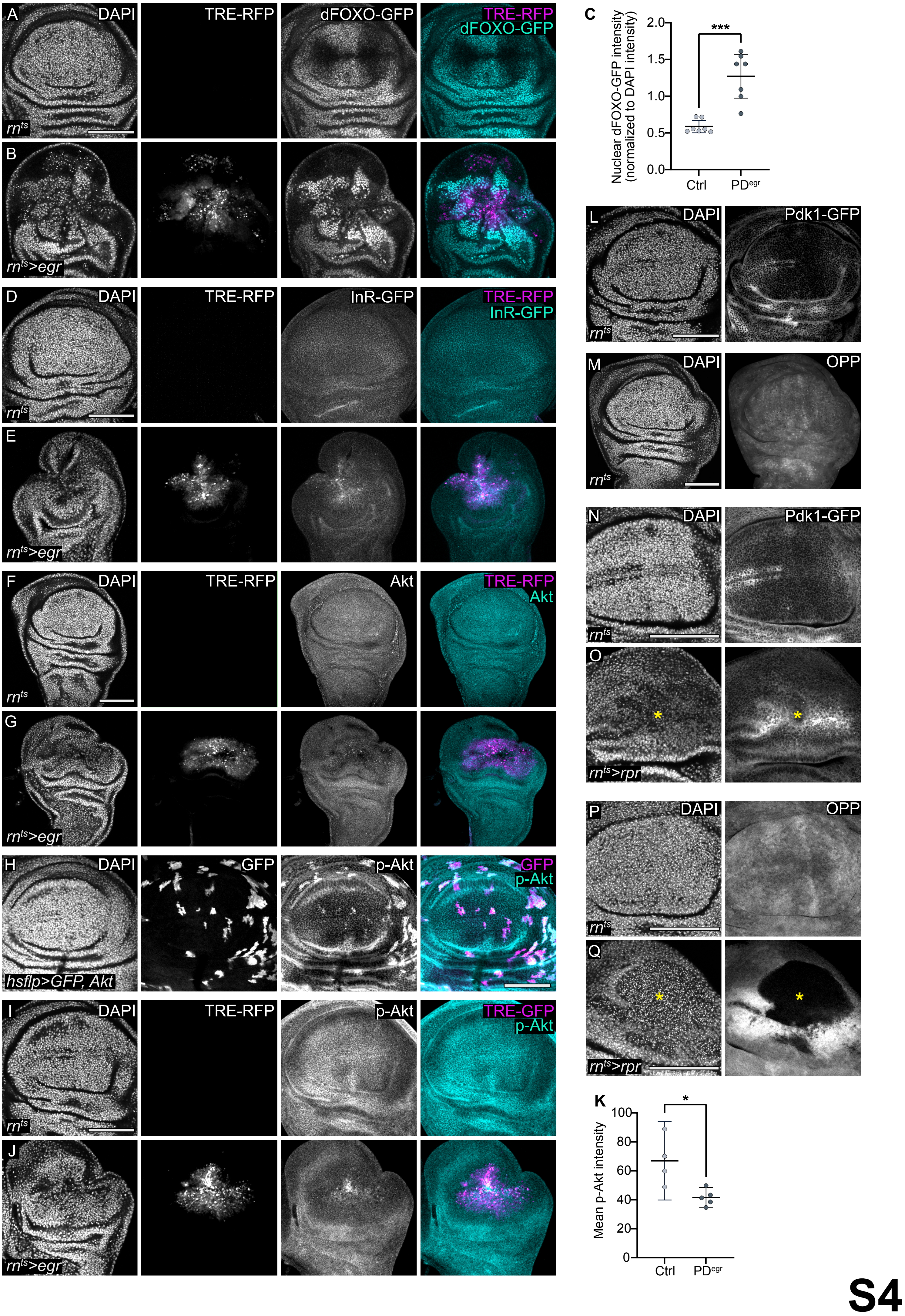

A DAPI TRE-RFP dFOXO-GFP TRE-RFP dFOXO-GFP
rnts
B
rnts>egr
C
Nuclear dFOXO-GFP intensity (normalized to DAPI intensity)
2.0 1.5 1.0 0.5 0.0
***
Ctrl PDegr
D DAPI TRE-RFP InR-GFP TRE-RFP InR-GFP
rnts
E
rnts>egr
F DAPI TRE-RFP Akt TRE-RFP Akt
rnts
G
rnts>egr
H DAPI GFP p-Akt GFP p-Akt
hsflp>GFP, Akt
I DAPI TRE-RFP p-Akt TRE-GFP p-Akt
rnts
J
rnts>egr
K
Mean p-Akt intensity
100 80 60 40 20 0
*
Ctrl PDegr
L DAPI Pdk1-GFP
rnts
M DAPI OPP
rnts
N DAPI Pdk1-GFP
rnts
O
rnts>rpr
P DAPI OPP
rnts
Q
rnts>rpr
S4

Figure S4. Insulin/PI3K/Akt signaling is low in the proliferative domain.

A,B. Expression of dFOXO-GFP (cyan or grey) in control (A) and *egr*-expressing discs (B). TRE-RFP visualizes JNK-pathway activity (magenta or grey) and DAPI visualizes nuclei (grey). dFOXO-GFP expression is visualized using BDSC 59766 fly line.

C. Mean nuclear dFOXO-GFP intensity quantified in the pouch of control discs or the proliferative domain of *egr*-expressing discs (PD^{egr}). Mean and 95% CI are shown and statistical significance was tested using the two-tailed Mann-Whitney test, p-value = 0.0006 (control: n=7, *egr*-expressing disc: n=7).

D-G. Control (D, F) and *egr*-expressing wing discs (E, G), expressing an Insulin receptor tagged with GFP (InR-GFP) (D, E) or stained for Akt (F, G) (cyan or grey). TRE-RFP visualizes JNK-pathway activity (magenta or grey). Discs were stained with DAPI to visualize nuclei.

H. Anti-phospho-Akt (S505) staining (cyan or grey) to test antibody specificity in UAS-Akt-overexpressing clones marked by co-expression of UAS-GFP (magenta or grey) in the wing imaginal disc. Discs were stained with DAPI to visualize nuclei.

I, J. Control (I) and *egr*-expressing wing discs (J), stained for phospho-Akt (S505) (cyan or grey). TRE-RFP visualizes JNK-pathway activity (magenta or grey). Discs were stained with DAPI to visualize nuclei.

K. Quantification of mean p-AKT intensity in the control pouch and proliferative domain of *egr*-expressing wing discs. Mean and 95% CI are shown and statistical significance was tested using a two-tailed Unpaired t-test , p-value =0.0156. (Control; n=4, *egr*-expressing discs; n=5).

L. Expression of Pdk1-GFP in the control wing imaginal disc corresponding to pro-apoptotic *hid*-expressing discs shown in **Fig4.K**. DAPI visualizes nuclei.

M. Protein synthesis visualized by OPP incorporation in the control wing imaginal disc corresponding to *hid*-expressing discs shown in **Fig4.L**. DAPI visualizes nuclei.

N, O. Expression of Pdk1-GFP in the control and pro-apoptotic gene Reaper (*rpr*) expressing discs. The yellow asterisk marks the damage or *rpr*-expressing region. DAPI visualizes nuclei.

P, Q. Protein synthesis visualized by OPP incorporation in the control and pro-apoptotic gene Reaper (*rpr*) expressing discs. The yellow asterisk marks the damage or *rpr*-expressing region. DAPI visualizes nuclei.

Scale bar: 100 μm.

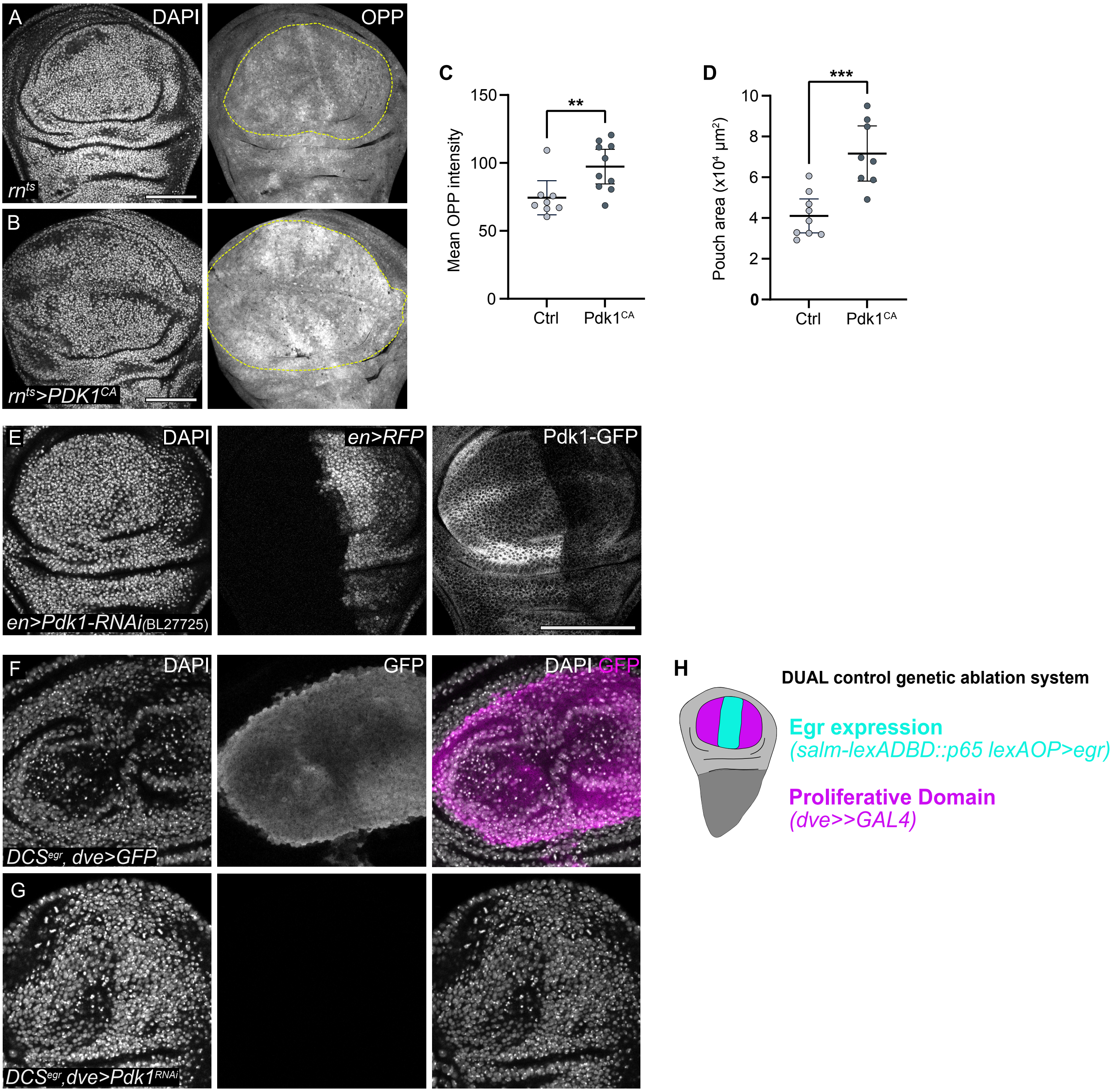

A
DAPI
OPP
rn^ts
B
rn^ts>PDK1^CA
C
Mean OPP intensity
150
100
50
0
**
Ctrl
Pdk1^CA
D
Pouch area (x10^4 µm^2)
10
8
6
4
2
0
***
Ctrl
Pdk1^CA
E
DAPI
en>RFP
Pdk1-GFP
en>Pdk1-RNAi(BL27725)
F
DAPI
GFP
DAPI GFP
DCS^egr, dve>GFP
G
DCS^egr,dve>Pdk1^RNAi
H
DUAL control genetic ablation system
Egr expression
(salm-lexADBD::p65 lexAOP>egr)
Proliferative Domain
(dve>>GAL4)

S5

Figure S5. Pdk1-GFP is necessary and sufficient to drive protein translation in the regenerative domain.

A, B. Protein synthesis visualized by OPP incorporation in control (A) and discs expressing a constitutively active UAS-Pdk1 (B) for 24 h under the control of *rn*-GAL4. Discs were stained with DAPI to visualize nuclei. The dotted white line marks the border of the pouch using tissue fold landmarks.

C. Quantification of mean OPP intensity in the pouch of control and *Pdk1*^{CA}-expressing wing discs. Mean and 95% CI are shown and statistical significance was tested using two-tailed Mann-Whitney test. p-value = 0.0062. (Control; n=8, *Pdk1*^{CA}-expressing discs; n=10).

D. Quantification of the pouch area in control and *Pdk1*^{CA}-expressing wing discs. Mean and 95% CI are shown and statistical significance was tested using two-tailed Unpaired t-test. p-value = 0.0003. (Control; n=9, *Pdk1*^{CA}-expressing discs; n=8).

E. Pdk1-GFP expression in the posterior compartment of the wing imaginal disc following Pdk1 knockdown using *en*-GAL4 to test the efficiency of Pdk1-RNAi line (BL27725). *en*-GAL4 which also drives expression of UAS-RFP expression to mark the posterior compartment. DAPI stains nuclei.

F,G. A DUAL Control genetic ablation system (DCS) was used to manipulate gene expression in the proliferative domain. Early third instar (L3) larvae were heat shocked at 37°C for 75min and dissected after 24 h. A single heat shock activates both ablation in the *salm* domain (tracked by pyknotic nuclei and MMP-1 upregulation) and genetic manipulation in the proliferative domain via *dve*-GAL4 (tracked by UAS-GFP co-expression in F). Dve-GAL4 expresses in the pouch and the proximal pouch fold, allowing the use of these morphological landmarks to approximate the dve-GAL4 domain that drives expression of *Pdk1-RNAi (G)*.

H. A simplified schematic representing DUAL control genetic ablation system (DCS). The genetically ablated region expresses *egr* under the control of *salm-lexADBD::p65 lexAOP* (marked in cyan), while *dve*-GAL4 is used to manipulate gene expression in the proliferative domain (magenta) surrounding the *egr* expressing region.

Scale bar: 100 µm. Fluorescence intensities are reported as arbitrary units.

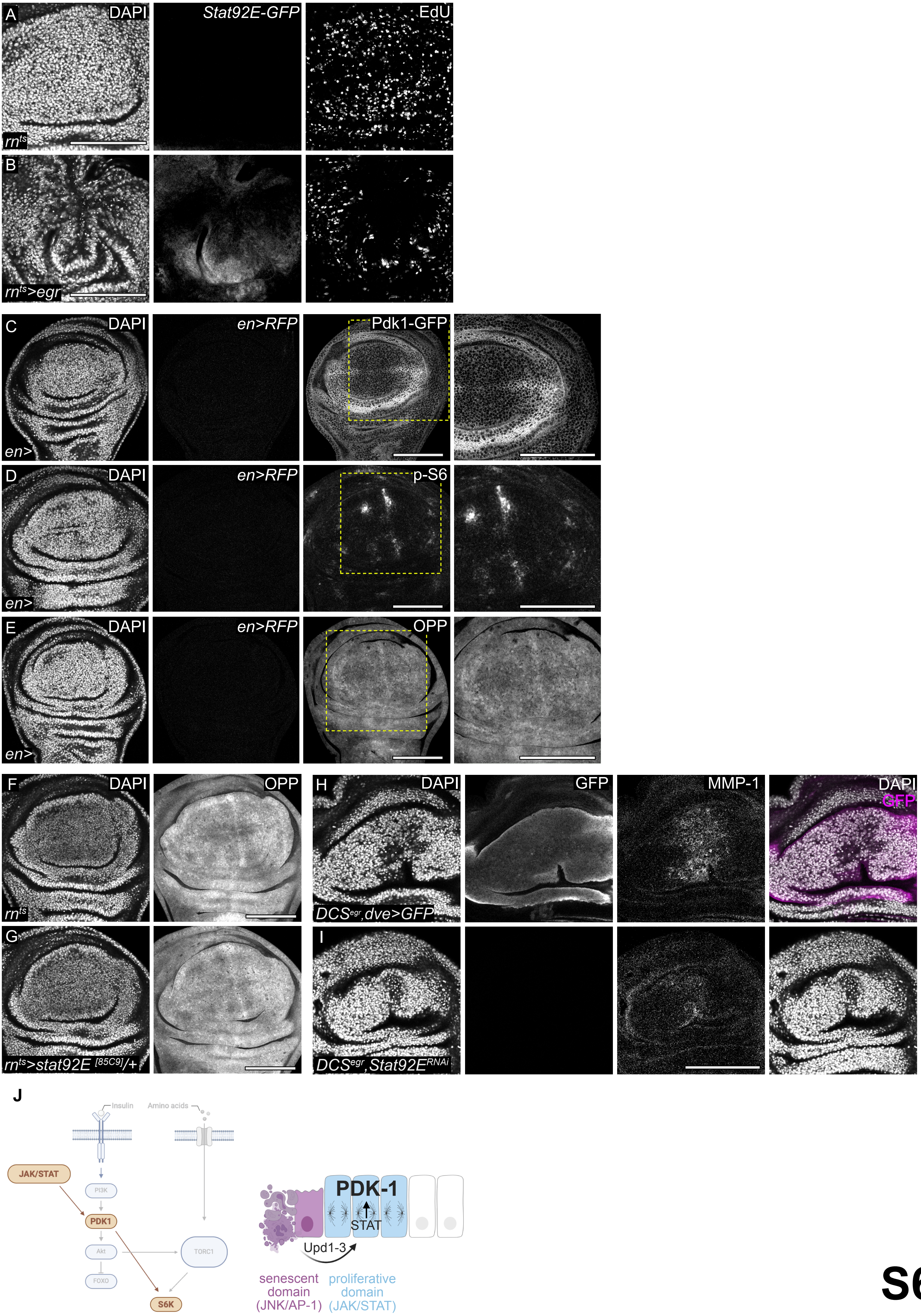

A
DAPI
Stat92E-GFP
EdU
rnts
B
rnts>egr
C
DAPI
en>RFP
Pdk1-GFP
en>
D
DAPI
en>RFP
p-S6
en>
E
DAPI
en>RFP
OPP
en>
F
DAPI
OPP
rnts
G
rnts>stat92E [85C9]/+
H
DAPI
GFP
MMP-1
DAPI
GFP
DCSegr,dve>GFP
I
DCSegr,Stat92ERNAi
J
Insulin
Amino acids
JAK/STAT
PI3K
PDK1
Akt
FOXO
TORC1
S6K
PDK-1
STAT
Upd1-3
senescent domain (JNK/AP-1)
proliferative domain (JAK/STAT)

S6

Figures S6. Pdk1 is regulated by JAK/STAT signaling

A, B. Control and *egr*-expressing discs stained for EdU to visualize DNA replication in discs also co-expressing a *Stat92E-GFP* activity reporter. Discs were also stained with DAPI to visualize nuclei. EdU intensity in *Stat92E-GFP* reporter-expressing domain was previously quantified (Jaiswal, Egert et al. 2023).
C, D, E. Corresponding control discs to the wing imaginal discs expressing UAS-STAT92E in the posterior compartment under the control *en*-GAL4 in **Fig 6.C, E and G**. Control discs were assayed for expression of Pdk1-GFP (C), levels of p-S6 (D), and protein synthesis using OPP incorporation assays (E). *en*-GAL4 also drives expression of UAS-RFP and DAPI staining visualizes nuclei. Yellow dotted box marks the inset region.
F,G. Protein synthesis visualized by OPP incorporation in wing discs that were either wild type (F) or heterozygous mutant for the $\textit{Stat92E}^{\textit{85C9}}$ null allele (G). DAPI staining visualizes nuclei.
H, I. Corresponding DAPI and MMP-1 stainings, as well as UAS-GFP expression of wing imaginal discs expressing the DUAL Control genetic ablation system (DCS) for images shown in **Fig 6.P and Q**. Early third instar (L3) larvae were heat shocked at 37°C for 75min and dissected after 40 h. A single heat shock activates both ablation in the *salm* domain (tracked by pyknotic nuclei and MMP-1 upregulation) and genetic manipulation in the proliferative domain via *dve*-GAL4 (tracked by UAS-GFP co-expression in H). Dve-GAL4 expresses in the pouch and the proximal pouch fold, allowing the use of these morphological landmarks to approximate the dve-GAL4 domain that drives expression of *stat92E-RNAi (I)*. Nuclei were visualized by DAPI staining in control (H) and *Stat92E* knockdown (I) wing imaginal disc.
J. A model of the JAK/STAT-Pdk1-S6K axis active in proliferating cells of *egr*-expressing discs. Unpaired ligands provided by senescent-like cells in the high JNK signaling domain activating JAK/STAT in the surrounding proliferative domain, leading to Pdk1 upregulation and insulin-independent growth.

Scale bar: 100 µm. Fluorescence intensities are reported as arbitrary units.

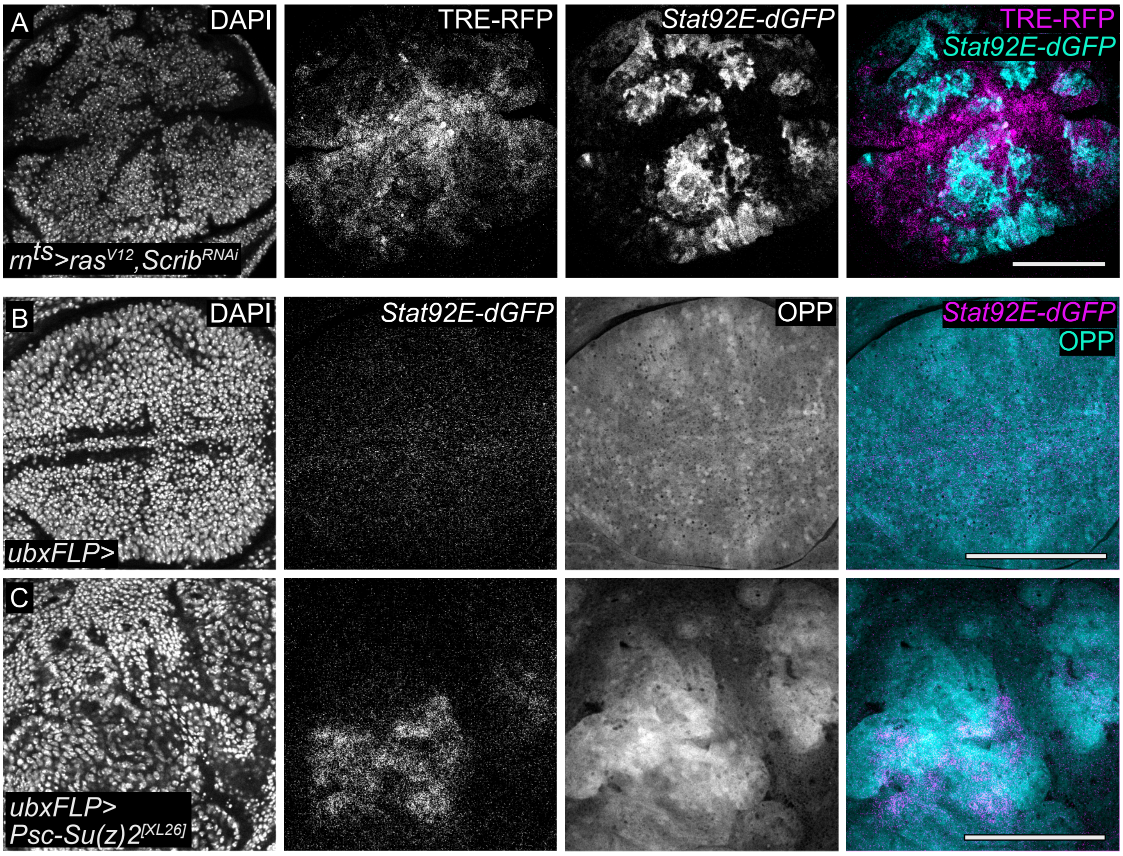
A
DAPI
TRE-RFP
Stat92E-dGFP
TRE-RFP
Stat92E-dGFP
rnts>rasV12,ScribRNAi
B
DAPI
Stat92E-dGFP
OPP
Stat92E-dGFP
OPP
ubxFLP>
C
ubxFLP>
Psc-Su(z)2[XL26]

S7

Figures S7. Protein translation and JAK/STAT signaling are linked in tumor growth

A. Wing disc expressing *Ras^{V12}, scrib-RNAi* for 44h starting at Day6 AED. The disc also expresses the JAK/STAT activity reporter *Stat92E-dGFP* (cyan or grey) and JNK/AP1 reporter TRE-RFP (magenta or grey). DAPI staining visualizes nuclei.

B, C. Control wing disc (B) and wing disc with mosaic clones mutant for the Polycomb family genes *Psc-Su(z)2^{XL26}* (GFP negative cells, C). Protein synthesis visualized by OPP incorporation (cyan or grey) and JAK/STAT activity assessed using *Stat92E-dGFP* reporter (magenta or grey). DAPI staining visualizes nuclei.

Scale bar: 100 µm.

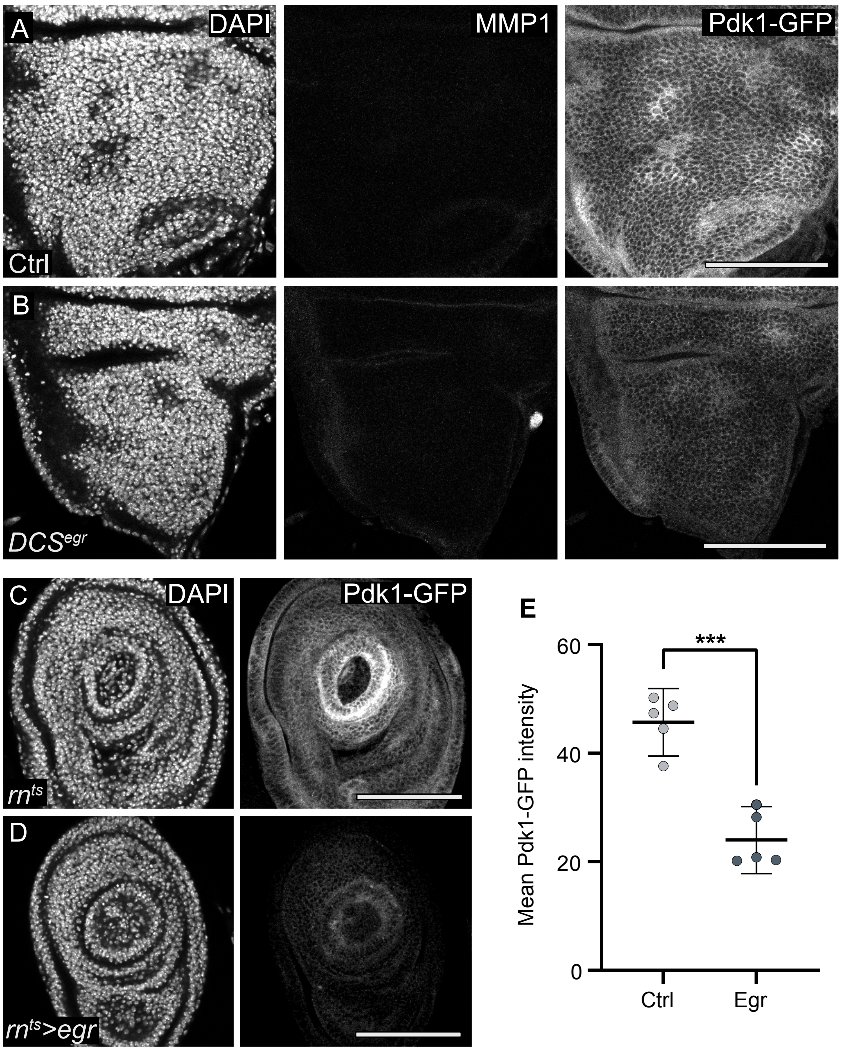
A
DAPI
MMP1
Pdk1-GFP
Ctrl
B
DCS^egr
C
DAPI
Pdk1-GFP
rn^ts
D
rn^ts>egr
E
***
60
40
20
0
Mean Pdk1-GFP intensity
Ctrl
Egr

S8

Figures S8. Pdk1 downregulation in peripheral discs correlates with systemic growth restriction

A, B. Expression of Pdk1-GFP in the notum of control (A) and *egr*-expressing discs in discs expressing the DUAL control genetic ablation system driving *egr*-expression (B). JNK/AP1 activation assessed by MMP1 staining. DAPI staining visualizes nuclei.

C, D. Expression of Pdk1-GFP in the leg imaginal disc, dissected from larvae with control (c) or *egr*-expressing (D) wing imaginal discs expressed using the *rn*-GAL4 driver.

E. Quantification of mean Pdk1-GFP intensity in the leg imaginal disc, dissected from larvae with control (C) or *egr*-expressing (D) wing imaginal discs. Statistical significance was tested using the two-tailed Unpaired t-test, p-value = 0.0001 (control: n=5, experiment: n=5).

Table S1 *Drosophila* strains

Genotype	Source
Dilp8-GFP	BDSC: 33079
ImpL2-GFP	BDSC: 59778
UAS-ImpL2-3xHA	Fly-ORF F001712
Upd3.1-3 LacZ	Ishwar Hariharan lab
dFOXO-GFP	BDSC: 59766 (FigS3.1C, D ; Fig S4 A, B)
dFOXO-GFP	BDSC 38644 (Fig 2D, E ; Fig4E, F)
salm-GAL4 / CyO,ubi-GFP	FBti0002632
tub-GAL80[ts], UAS-egr	Iswar Hariharan
UAS-hid/CyO	Gines Morata
UAS-rpr	BDSC: 5823
en2.4-GAL4, UAS-mRPF.NLS/CyO	BDSC: 30557
Tret1-GFP	BDSC: 66367
Pdk1-GFP	BDSC: 59836
InR-eGFP	Hong Xu lab<br>DOI: 10.7554/eLife.49309
tGPH-GFP	Yohanns Bellaiche
UAS-Akt1	BDSC: 8192
CG5535-GFP	BDSC: 64096
UAS-GFP^{S56T}	BDSC: 1521
rn$^{GAL4\text{-}DeltaS}$*, tubGAL80*^{ts}	BDSC: 8142, 7012
rn$^{GAL4\text{-}5}$*, UAS-egr, tubP-GAL80*^{ts}	Iswar Hariharan
10xStat92E-dGFP	Erica Bach<br>PMID: 17008134
10xStat92E-GFP	Erica Bach<br>PMID: 17008134
UAS-Stat92E.ORF.3xHA	Fly-ORF F000750<br>PMID: 23583758
'TRE-RFP': TRE-DsRed.T4	Dirk Bohmann<br>PMID: 22509270
upd3.1-3-LacZ	Iswar Hariharan
UAS-dPdk1	Hugo Stocker
UAS-dPdk1(A467V)	Hugo Stocker<br>https://doi.org/10.1073/pnas.011318098
dPdk1(5)ΔEP	Hugo Stocker lab<br>https://doi.org/10.1073/pnas.011318098
yw, hsflp[122]; hs-p65::zip, lexAOp-egrNI /CyO-GFP; salm-zip::LexADBD, Dve>stop>GAL4/TM6c	Robin Harris<br>PMID: 32490812
UAS-Pdk1-RNAi	BDSC: 27725 (Fig.5L, M: Fig. S5G: FigS6.F, G)
UAS-Pdk1-RNAi	BDSC: 34936 (used additionally to validate *Pdk1-GFP* line, BDSC: 59836)
UAS-Pdk1-RNAi	BDSC: 36071 (used additionally to validate *Pdk1-GFP* line, BDSC: 59836)
UAS-stat92E RNAi	BDSC: 33637
stat 92E [85c9]	Erica Bach
UAS-Ras^{V12}*, scrib-RNAi*	FBtp0001705, VDRC: 105412

ubxFLP; FRT42D ubi-mRFP	FBti0150334, FBti0147338
FRT42D Psc-Su(z)2[XL26]	Rongwen Xi<br>PMID: 20439432
w^{1118}	

Table S2 Immunohistochemistry reagents

Reagent	Source	
Mouse polyclonal anti-β-Galactosidase (1:1000)	Promega	Cat. #: Z3782
Mouse monoclonal anti-MMP-1 (1:30)	Developmental Studies Hybridoma Bank	Cat. #: 3A6B4
Mouse monoclonal anti-MMP-1 (1:30)	Developmental Studies Hybridoma Bank	Cat. #: 3B8D12
Mouse monoclonal anti-MMP-1 (1:30)	Developmental Studies Hybridoma Bank	Cat. #: 5H7B11
Mouse monoclonal anti-Nubbin (1:100)	Developmental Studies Hybridoma Bank	Cat. #: 2D4
Rat monoclonal anti-elav (1:50)	Developmental Studies Hybridoma Bank	Cat. #: 7E8A10
Rabbit polyclonal anti-CG1139 (1:200)	Susumu Hirabayashi, Imperial College London Faculty of Medicine.	https://pubmed.ncbi.nlm.nih.gov/32938923/
Rabbit polyclonal anti-p-AKT (S505) (1:500)	Cell Signaling	Cat. #: 4084s
Rabbit polyclonal anti-p-AKT (S473) (1:500)	Cell Signaling	Cat. #: 4060s
Rabbit polyclonal anti-AKT (1:500)	Cell Signaling	Cat. #: 9272s
Rat monoclonal anti-RFP (1:1000)	Chromotek	Cat. #: 5F8
Rabbit polyclonal anti-Drosophila p-S6 (1:200)	Aurelio Telemann, DKFZ	https://doi.org/10.1016/j.devcel.2017.07.019
Rabbit polyclonal anti-Ref(2)p	Abcam	Cat. #: Ab178440
Rabbit anti-cleaved Dcp-1	Cell Signaling	Cat. #: 9578s
Rabbit monoclonal anti-GFP	Invitrogen	Cat. #: G10362
Goat anti-mouse IgG Alexa Flour 488	Invitrogen	Cat. #: A-11001
Goat anti-rabbit IgG Alexa Flour 488	Invitrogen	Cat. #: A-11008
Goat anti-mouse IgG Alexa Flour 555	Invitrogen	Cat. #: A-21422
Goat anti-rat IgG Alexa Flour 555	Invitrogen	Cat. #: A-21434
Goat anti-mouse Alexa Flour 647	Invitrogen	Cat. #: A-21235
Goat anti-rabbit Alexa Flour 647	Invitrogen	Cat. #: A-21244
HRP-conjugated anti-rabbit IgG	Promega	Cat. #: W4011
Click-iT Plus EdU Alexa Fluor 647 Imaging Kit	Invitrogen	Cat. #: C10340
Phalloidin 488	Invitrogen	Cat #: A12379
Phalloidin 647	Invitrogen	Cat #: A22287
DAPI	Sigma Aldrich	Cat #: D9564
CellEvent Senescence Green Detection Kit	Invitrogen	Cat. #: C10850
Rapamycin	Thermo Fisher Scientific	Cat. #: J62473.MC
Click-iT Plus OPP Alexa Fluor 647 Imaging Kit	Invitrogen	Cat. #: C10458
Leptomycin	Sigma-Aldrich	Cat. #: L2913
Nile Red	Invitrogen	Cat. #: N1142
Trichlormethan/ Chloroform	Roth	Cat. #: 7331.2
2-Propanol	Merck	CAS-Nr. 67-63-0

TRIzol™ Reagent	Thermo Fisher	Cat. #: 15596026
Random Hexamer Primer	Thermo Fisher	Cat. #: N8080127
DNase I, RNase-free	Thermo Fisher	Cat. #: ENO521
RevertAid Reverse Transcriptase	Thermo Fisher	Cat. #: EP0441
RiboLock RNase Inhibitor	Thermo Fisher	Cat. #: EO0381
dNTP-Set	Thermo Fisher	Cat. #: R0182
Blue S'Green qPCR Kit Separate ROX	Biozym	Art. #: 331416
TLC Silica gel 60, 50 Glass plates 10 x 20 cm	Merck	Cat. #: 10526
Methanol	Roth	Art. #: 8388.6
n-Hexan	Roth	Art. #: 7339.1
Diethylether	Roth	Art. #: 5920.2
Sulfuric acid	Sigma-Aldrich	Art.#: 339741
Ammonium heptamolybdate tetrahydrate	Merck	Cat. #: 101180
Cerium (IV) sulfate hydrate, complex	Sigma-Aldrich	Art.#: 945512345
2-Mercaptoethanol	Sigma Aldrich	Art.#:M6250
Mini-PROTEAN TGX gels	Bio-Rad	Cat. #: 4561083
Turbo Midi 0.2 µM Nitrocellulose Transfer Packs	Bio-Rad	Cat. #: 1704159
Bio-Rad Trans-Blot Turbo Transfer System	Bio-Rad	Cat. #: 1704150
SuperSignal West Femto Maximum Sensitivity Substrate	Thermo Fisher	Cat. #: 34095

File S1 Quantification workflows and FIJI macros

Quantification Fig.1D

1: Select pouch slices while excluding the peripodium.
2: Generate a TRE-RFP mask to define the high JNK region.
3: Create 10 μm bands inside and outside the TRE-RFP mask boundary.
4: Generate a DAPI mask to identify nuclei.
5: Use the FIJI Binary AND function to create DAPI masks for each 10 μm band.
6: Measure EdU intensity within the DAPI mask for each band

Macro-1D(a). Generating TRE-RFP Mask
To generate the TRE Mask, slices from the Z-stack were first duplicated, excluding slices containing peripodium cells. These duplicated slices were then projected to maximum intensity, focusing on Channel 3, which contains the TRE-RFP staining. The workflow was executed as follows:

```
run("Duplicate...", "duplicate channels=3");
run("Z Project...", "projection=[Max Intensity]");
run("Gaussian Blur...", "sigma=2");
setAutoThreshold("Default");
//run("Threshold...");
//setThreshold(0, 66);
run("Convert to Mask");
run("Save");
```

First, the Channel 3 (TRE-RFP channel) slices were duplicated. Next, a maximum intensity projection was applied to these slices. A Gaussian blur with a sigma value of 2 was then applied to smooth the image. The setAutoThreshold function with the "Default" setting was used to automatically determine the threshold. The image was then converted to a binary mask using the Convert to Mask function, and the resulting mask was saved.
This process ensured a consistent and accurate generation of the TRE mask, allowing for precise quantification in subsequent analyses.

Macro-1D(b). Generating DAPI Mask
To generate the DAPI Mask, slices from the Z-stack were first duplicated, excluding peripodium cells. These duplicated slices were then projected to maximum intensity, focusing on the DAPI Channel (Channel 1). The workflow was executed as follows:

```
run("Duplicate...", "duplicate channels=1");
run("Z Project...", "projection=[Max Intensity]");
run("Enhance Local Contrast (CLAHE)", "blocksize=127 histogram=256 maximum=3 mask=*None*
fast_(less_accurate)");
setAutoThreshold("Otsu");
//run("Threshold...");
//setThreshold(0, 86);
setOption("BlackBackground", false);
run("Convert to Mask");
```

First, the Channel 1 (DAPI channel) slices were duplicated. Next, a maximum intensity projection was applied to these slices. The local contrast of the image was enhanced using the CLAHE method with a block size of 127, histogram bins of 256, and a maximum slope of 3. The setAutoThreshold function with

the "Otsu" setting was used to automatically determine the threshold. The BlackBackground option was set to false to ensure proper mask generation. Finally, the image was converted to a binary mask using the Convert to Mask function. This process ensured the consistent and accurate generation of the DAPI mask, allowing for precise quantification in subsequent analyses.

Quantification Fig.1G

Undamaged control disc:

- Manually mark the pouch, hinge, and notum regions using wing disc folds as a reference.
- Measure OPP intensity separately in these three regions.

Egr-expressing disc:

- Measure OPP intensity in the high JNK region, proliferative domain, and notum region.
- High JNK Mask Creation: Follow specific macro steps to generate the mask.
- Proliferative Domain: Define this region using a 20 µm band generated from the high JNK mask boundary.

Macro-1G(a). Generating TRE-RFP Mask

Slices from the egr expressing discs were selected and duplicated, excluding peripodium cells. The following macro was applied to generate a TRE mask. File name Series14 (S14) used as an example.

```
macro "Manual File Name Input" {
   // Prompt for the custom file name part
   customName = getString("Enter custom name part (e.g., S14):", "S14");
   // Define the base folder path, Change the file location as you wish
   saveFolder = "G:/Core/maya/Manuscript/Quantifications/Q1_OPP in 3 
Region/Quantification_Opp_Eiger/Series14/";
   // Duplicate the slices (the slices=4-12 option commented out, uncomment if needed)
   // run("Duplicate...", "duplicate slices=4-12");
   run("Duplicate...", "duplicate");
   // Save the first duplicated image
   saveAs("Tiff", saveFolder + customName + "_Selected slices.tif");
   // Duplicate specific channel (channel 3 in this case)
   run("Duplicate...", "duplicate channels=3");
   // Save the second duplicated image
   saveAs("Tiff", saveFolder + customName + "_Selected slices_TRE channel-1.tif");
   // Perform Z projection
   run("Z Project...", "projection=[Max Intensity]");
   // Save the Z projection image
   saveAs("Tiff", saveFolder + "MAX_" + customName + "_Selected slices_TRE channel-1_Max Project.tif");
   // Duplicate the image
   run("Duplicate...", " ");
   // Select the image by title
   selectImage("MAX_" + customName + "_Selected slices_TRE channel-1_Max Project-1.tif");
   // Apply Gaussian Blur
   run("Gaussian Blur...", "sigma=4");
   // Apply auto threshold
   setAutoThreshold("Moments");
   // Optional: Set specific threshold values (uncomment if needed)
   // setThreshold(0, 47);
   // Set background option
```

```
  setOption("BlackBackground", false);
  // Convert to mask
  run("Convert to Mask");
  // Apply dilate operation
  run("Dilate")
}
```

First, slices from the Z-stack were duplicated, excluding peripodium cells. A custom name for the file was input to organize the output files. The selected slices were duplicated, and the duplicate image was saved. Next, Channel 3, containing the TRE-RFP staining, was isolated and saved. These slices were then projected to maximum intensity using Z projection, and the resulting image was saved.
The maximum intensity projection image was duplicated, and a Gaussian blur with a sigma of 4 was applied to smooth the image and reduce noise. Auto-thresholding was performed using the "Moments" setting to determine the optimal threshold value for mask generation. The background option was set to false to ensure proper mask creation, and the image was converted to a binary mask using the Convert to Mask function. Finally, a dilation operation was applied to refine the mask. This process ensured accurate generation of the TRE mask for precise quantification in subsequent analyses.

Quantification Fig.1L

Quantification of EdU Area per DAPI Area:

1. Generate a DAPI Mask for the notum region.
2. Generate an EdU Mask for the same region.
3. Calculate the EdU-positive area as a proportion of the DAPI area, serving as a proxy for the number of proliferating cells.

Macro-1L(a). Generating DAPI Mask

Selected a single slice from Z-stack containing maximum number of nuclei, identified with DAPI staining. And applied the following work flow in the macro to generate a DAPI mask.

```
run("Duplicate...", "duplicate channels=1");
run("Subtract Background...", "rolling=100");
run("Enhance Local Contrast (CLAHE)", "blocksize=127 histogram=256 maximum=3 mask=*None*
fast_(less_accurate)");
setAutoThreshold("Moments dark");
//run("Threshold...");
//setThreshold(97, 255);
setOption("BlackBackground", false);
run("Convert to Mask");
run("Invert LUT");
//saveAs("Tiff", "Location for saving the file");
run("Duplicate...", " ");
run("Invert");
run("Save");
```

First, Channel 1 (DAPI channel) was duplicated. The background was subtracted using a rolling ball radius of 100 pixels. Local contrast enhancement was performed using the CLAHE method with a block size of 127, histogram bins of 256, and a maximum slope of 3. The setAutoThreshold function with the "Moments dark" setting was used to automatically determine the threshold. The BlackBackground option was set to false to ensure proper mask generation, and the image was converted to a binary mask using

the Convert to Mask function. The lookup table (LUT) was inverted for correct visualization. The image was duplicated and inverted to create the final mask. This process ensured accurate generation of the DAPI mask for precise quantification in subsequent analyses.

Quantification Fig.1M

Quantification of DNA Replication Speed:
1. Generate an EdU Mask for the notum region.
2. Measure EdU Intensity within the EdU-positive area.
3. Use EdU intensity as a proxy for the speed of DNA replication.

Macro-1M(a). Generating EdU Mask
A single slice from the Z-stack, containing the maximum number of nuclei identified with DAPI staining, was selected. The following workflow was applied to generate an EdU mask:

```
run("Duplicate...", " ");
run("Gaussian Blur...", "sigma=2");
setAutoThreshold("Moments dark");
//run("Threshold...");
//setThreshold(28, 255);
setOption("BlackBackground", false);
run("Convert to Mask");
run("Create Selection");
roiManager("Add");
```

First, the selected slice was duplicated. A Gaussian blur with a sigma value of 2 was applied to smooth the image. The setAutoThreshold function with the "Moments dark" setting was used to automatically determine the threshold. The BlackBackground option was set to false to ensure proper mask generation, and the image was converted to a binary mask using the Convert to Mask function. A selection was created from the mask, and this region of interest (ROI) was added to the ROI Manager. This process ensured accurate generation of the EdU mask for precise quantification in subsequent analyses.

Quantification Fig.2F

Quantification of Nuclear dFOXO Intensity:
1. Select a single slice with the maximum number of nuclei in the notum.
2. Create a DAPI mask for the notum from the selected slice.
3. Measure the mean nuclear dFOXO intensity using this mask.

Macro-2F (a). Generating DAPI Mask
A single slice from the Z-stack, containing the maximum number of notum nuclei identified with DAPI staining, was selected. The following workflow was applied to generate a DAPI Mask

```
run("Duplicate...", "duplicate channels=1");
run("Subtract Background...", "rolling=100");
run("Gaussian Blur...", "sigma=1");
run("Enhance Local Contrast (CLAHE)", "blocksize=127 histogram=256 maximum=3 mask=*None*
fast_(less_accurate)");
setAutoThreshold("Otsu dark");
```

```
//run("Threshold...");
//setThreshold(112, 255);
setOption("BlackBackground", false);
run("Convert to Mask");
run("Save");
```

A duplicate of the original image was created, selecting channel 1(DAPI channel) for processing. Background subtraction was performed using a rolling ball radius of 100 to reduce uneven illumination. A Gaussian blur with a sigma of 1 was applied to smooth the image. Contrast enhancement was conducted using CLAHE (block size = 127, histogram bins = 256, maximum slope = 3) to improve local contrast. Automatic thresholding was applied using the Otsu method to segment the image. The image was then converted into a binary mask with a black background setting disabled, and the final processed mask was saved for further analysis.

Quantification Fig.2Q

Quantification of OPP Intensity in the Eye Disc:

1. Select slices with the maximum number of cells in the eye disc.
2. Generate a tissue mask using the DAPI channel.
3. Measure OPP intensity in the eye disc using the generated tissue mask.

Macro-2Q(a). Generating Tissue Mask

A single slice from the Z-stack with the maximum number of nuclei in the larger lobe of the eye disc, identified using DAPI staining, was selected. The following workflow was applied to generate a Tissue mask outlining the region with cells:

```
run("Duplicate...", "duplicate channels=1");
run("Gaussian Blur...", "sigma=3");
setAutoThreshold("Moments dark");
//run("Threshold...");
//setThreshold(53, 255);
setOption("BlackBackground", false);
run("Convert to Mask");
run("Dilate");
run("Dilate");
```

First, the slice was duplicated, focusing on Channel 1, which contains the DAPI staining. Gaussian blur with a sigma of 3 was applied to smooth the image and reduce noise. Auto-thresholding was performed using the "Moments dark" setting to determine the optimal threshold value for mask generation. The background option was set to false to ensure proper mask creation, and the image was converted to a binary mask using the Convert to Mask function. Finally, two dilation operations were applied to refine and slightly enlarge the mask, ensuring all relevant cell regions were included. This process ensured accurate generation of the Tissue mask, outlining the regions with cells for subsequent analyses.

Quantification- Fig-2T

Quantification of DNA Replication Speed:

1. Select slices from the z-stack with the maximum number of cells.
2. Create an EdU mask using the EdU channel.

3. Measure EdU intensity in the EdU-positive area using the EdU mask. This measurement serves as a proxy for the speed of DNA replication.

Macro-2T(a). Generating EdU Mask

To generate the EdU mask for eye disc, a single slice from the Z-stack containing the maximum number of nuclei was selected using DAPI staining. The following workflow was applied:

```
// Prompt user to select an image file
//open(getFile("Select an image file"));
// Get the base name of the opened image (without extension)
inputTitle = replace(getTitle(), ".tif", "");
eduSliceTitle = inputTitle + "-EdU-Slice";
eduMaskTitle = inputTitle + "-EdU-Mask";
// Duplicate the selected channel (Channel 4)
run("Duplicate...", "duplicate channels=4");
// Define save directory
saveDirectory = "G:/Core/maya/Manuscript/003_Quantifications/Q38_EdU_Eye and Leg disc/";
// Save duplicated image
saveAs("Tiff", saveDirectory + "Step2-EdU Slice/" + eduSliceTitle + ".tif");
// Duplicate the image again for processing
run("Duplicate...", "");
// Preprocessing steps
run("Subtract Background...", "rolling=50");
run("Gaussian Blur...", "sigma=1");
setAutoThreshold("Otsu dark");
// Apply mask conversion
setOption("BlackBackground", false);
run("Convert to Mask");
// Save the generated mask
saveAs("Tiff", saveDirectory + "Step3-EdU Mask/" + eduMaskTitle + ".tif");
// Close processed images
close();
close();
```

The selected image was processed to extract and analyze the EdU signal. First, channel 4 (EdU channel) of the image was duplicated to isolate the EdU signal. The duplicated image was saved in the designated directory as the EdU slice. A second duplicate was created for further processing. Preprocessing steps included background subtraction using a rolling ball radius of 50 to reduce noise and Gaussian blur (sigma = 1) to smooth the image. Automatic thresholding was applied using the Otsu method for segmentation. The resulting binary mask was generated with the black background setting disabled and saved in the designated directory as the EdU mask. Finally, processed images were closed.

Quantification Fig.3C, 3K, S3A and S3I

Lipid droplet analysis was performed using an automated image processing macro from Dark et.al. 2022 (https://doi.org/10.1016/j.xpro.2022.101230). The macro first prompted the user to select input and output directories. It then processed all .tif images in the input folder. For each image, a representative slice was selected, and background subtraction (rolling ball radius = 50) and contrast enhancement were applied. A region of interest (ROI) was manually defined, duplicated, and thresholded using the Li dark

method, followed by watershed segmentation to separate individual droplets. Lipid droplets were analyzed based on area, roundness and results were saved as .csv files. Proof images with overlaid measurements and scale bars were generated and saved. The process was repeated for three ROIs per image. Finally, all intermediate images were closed, and memory was cleared to optimize performance.

Quantification- Fig.3H

Quantification of ImpL2-GFP Intensity in High JNK Region:

1. Select pouch slices without peripodium and create a projection of the selected slices, ensuring consistency in the number of slices used.
2. Generate a Nubbin mask using Nubbin staining.
3. Measure ImpL2-GFP intensity inside the high JNK region of the damaged disc using the Nubbin mask.

Macro-3H(a). Generating Nubbin Mask

To generate the Nubbin mask, slices from the Z-stack were first duplicated, excluding peripodium cells. These duplicated slices were then projected to maximum intensity, focusing on Channel 3, which contains the Nubbin staining. The following workflow was applied:

```
run("Duplicate...", "duplicate channels=3");
run("Gaussian Blur...", "sigma=3");
setAutoThreshold("Moments dark");
//run("Threshold...");
//setThreshold(100, 255);
setOption("BlackBackground", false);
run("Convert to Mask");
run("Invert LUT");
run("Maximum...", "radius=6");
```

First, the slices were duplicated, focusing on Channel 3 (Nubbin channel) to isolate the Nubbin staining. A Gaussian blur with a sigma of 3 was applied to smooth the image and reduce noise. Automatic thresholding was performed using the "Moments dark" setting to determine the optimal threshold value for mask generation. The BlackBackground option was set to false to ensure proper mask creation, and the image was converted to a binary mask using the Convert to Mask function. The lookup table (LUT) was then inverted to complete the mask, and the Maximum filter with a radius of 6 was applied to enhance the mask by expanding the detected regions. This process ensured accurate generation of the Nubbin mask for precise quantification in subsequent analyses.

Quantification Fig-S3H

Quantification of upd3-LacZ Intensity in High JNK Region:

1. Select pouch slices without peripodium and create a projection of the selected slices, ensuring consistency in the number of slices used.
2. Generate a high JNK mask using TRE-RFP staining.
3. Measure Upd3-LacZ intensity inside the high JNK region of the damaged disc using the TRE-RFP mask.

Macro-S3H(a). Generating TRE-RFP Mask

To generate the TRE mask, slices from the Z-stack were first duplicated, excluding peripodium cells. These duplicated slices were then projected to maximum intensity, focusing on Channel 3, which contains the TRE-RFP staining. The following workflow was applied:

```
run("Z Project...", "projection=[Max Intensity]");
run("Duplicate...", "duplicate channels=3");
run("Gaussian Blur...", "sigma=2");
setAutoThreshold("IsoData dark");
//setThreshold(47, 255);
setOption("BlackBackground", false);
run("Convert to Mask");
run("Invert LUT");
```

First, the slices were projected using maximum intensity projection to enhance the visibility of the TRE-RFP signal. The projected slices were then duplicated, focusing on Channel 3. A Gaussian blur with a sigma of 2 was applied to smooth the image. Thresholding was performed with the "IsoData dark" setting, to determine the optimal threshold values. The BlackBackground option was set to false to ensure proper mask generation, and the image was converted to a binary mask using the Convert to Mask function. Finally, the lookup table (LUT) was inverted to complete the mask. This process ensured accurate generation of the TRE mask for precise quantification in subsequent analyses.

Quantification Fig.3O

Quantification of OPP Intensity in Undamaged and Egr-Expressing Damaged Discs:

1. For undamaged discs, manually select the wing pouch using wing folds as a reference and measure OPP intensity in the pouch.
2. For egr-expressing damaged discs, measure OPP intensity in the proliferative domain within a 20 µm region from the high JNK region, using the TRE-RFP mask.
3. The TRE-RFP mask is generated using the following macro.
4. The tissue mask for the proliferative domain is created using DAPI staining, with the following macro.

Similar logic is followed for **Fig5K, Fig6.O**

Macro-3O (a) Generating TRE-RFP Mask for damaged disc

To generate the TRE mask, slices from the Z-stack were first duplicated, excluding peripodium cells. These duplicated slices were then projected to maximum intensity, focusing on Channel 3, which contains the TRE-RFP staining. The following workflow was applied:

```
run("Z Project...", "projection=[Max Intensity]");
run("Gaussian Blur...", "sigma=2");
setAutoThreshold("Otsu dark");
//run("Threshold...");
//setThreshold(45, 255);
setOption("BlackBackground", false);
run("Convert to Mask");
run("Fill Holes");
run("Erode");
run("Erode");
run("Erode");
run("Save");
```

A maximum intensity projection was generated from the Z-stack images using Fiji. The resulting image was smoothed with a Gaussian blur (σ = 2) to reduce noise. An automatic thresholding method (Otsu, dark background) was applied to segment the structures of interest, followed by conversion to a binary mask. Small enclosed regions were filled, and three erosion steps were performed to refine the segmentation. The final processed mask was then saved for further analysis.
For undamaged wing discs, the pouch region was manually selected based on morphological landmarks, using the natural folds of the wing disc as a reference.
A 20-micron band was generated from the TRE-Mask edge to delineate the proliferative domain in egr-expressing damaged discs. This same 20-micron band was consistently used throughout the quantification to measure parameters within the proliferative domain.

Macro-3O (b) Generating Tissue Mask
To generate a tissue mask, slices from the Z-stack were first duplicated focusing on Channel 1, which contains the DAPI staining. The following workflow was applied:

```
run("Duplicate...", "duplicate channels=1");
run("Gaussian Blur...", "sigma=2");
setAutoThreshold("Moments dark");
//run("Threshold...");
//setThreshold(40, 255);
run("Convert to Mask");
run("Dilate");
run("Dilate");
run("Dilate");
run("Save");
```

DAPI channel was duplicated for processing. A Gaussian blur (σ = 2) was applied to reduce noise, followed by automatic thresholding using the Moments method (dark background) to segment the structures of interest. The image was then converted into a binary mask. To enhance the segmented features, three dilation steps were performed. The final processed mask was then saved for further analysis.

Quantification- Fig.4C

Quantification of p-S6 Intensity in the Proliferative Domain:

1. Generate a high JNK region TRE-RFP mask.
2. Create a 20 μm band around the TRE-RFP mask to define the proliferative domain.
3. Measure p-S6 intensity inside the 20 μm band.
4. Compare the measured p-S6 intensity in the proliferative domain of the damaged disc with the p-S6 intensity in the undamaged pouch.

Similar approach for quantification **Fig.3R, Fig.S3.1-U, Fig.S4K and Fig.5J**

Macro-4C(a) Generating TRE-RFP Mask for damaged disc
To generate the TRE mask, slices from the Z-stack were first duplicated, excluding peripodium cells. These duplicated slices were then projected to maximum intensity, focusing on Channel 3, which contains the TRE-RFP staining. The following workflow was applied

```
run("Z Project...", "projection=[Max Intensity]");
run("Gaussian Blur...", "sigma=2");
setAutoThreshold("Default dark");
//setThreshold(53, 255);
```

```
setOption("BlackBackground", false);
run("Convert to Mask");
run("Dilate");
run("Fill Holes");
run("Erode");
run("Erode");
run("Save");
```

A maximum intensity Z-projection was generated to combine image stacks into a single representative image. A Gaussian blur (σ = 2) was applied to reduce noise and smooth the image. Automatic thresholding using the Default (dark) method was applied to segment structures of interest. The image was then converted into a binary mask, followed by a dilation step to enhance feature boundaries. Holes within the segmented regions were filled, and two erosion steps were performed to refine the mask. The final processed image was saved for further analysis.

Quantification Fig.4G

Quantification of Nuclear dFOXO Intensity in the Nucleus:

1. Generate a DAPI mask to identify the nuclei.
2. Separate the nuclei in the proliferative domain by creating a 20 μm band around the TRE-RFP mask.
3. Generate a high JNK region TRE-RFP mask using the appropriate macro.
4. Measure nuclear dFOXO intensity within the identified nuclei in the proliferative domain using the DAPI mask and the 20 μm band.

Similar approach for quantification **Fig.S4C**

Macro-4G (a) Generating TRE-RFP Mask for damaged disc

To generate the TRE mask, slices from the Z-stack were first duplicated, excluding peripodium cells. These duplicated slices were then projected to maximum intensity, focusing on Channel TRE-RFP channel. The following workflow was applied

```
// Prompt user to select an image file
//open(getFile("Select an image file"));
// Get the title of the opened image (remove extension for consistency)
inputTitle = replace(getTitle(), ".tif", "");
// Duplicate the image
run("Duplicate...", "");
// Perform background subtraction
run("Subtract Background...", "rolling=120");
// Apply Gaussian blur
run("Gaussian Blur...", "sigma=3");
// Apply auto-thresholding using Otsu's method
setAutoThreshold("Otsu dark");
// Convert to mask
setOption("BlackBackground", false);
run("Convert to Mask");
// Process the mask: Fill Holes, Erode, and Dilate
run("Fill Holes");
run("Erode");
run("Erode");
run("Erode");
```

```
run("Dilate");
// Define save path dynamically
savePath = "G:/Core/maya/Manuscript/003_Quantifications/Q42_FOXO-GFP_PD_AC2108/Step4-TRE Mask/MAX_" + inputTitle + "-1.tif";
// Save the processed mask
saveAs("Tiff", savePath);
// Close the duplicated image
close();
// Close the original image
selectImage(inputTitle + ".tif");
close();
```

An image file was selected and duplicated for processing. Background subtraction was performed using a rolling ball radius of 120 to enhance contrast. A Gaussian blur (σ = 3) was applied to smooth noise before segmentation. Automatic thresholding with Otsu's method was used to create a binary representation, followed by conversion into a mask. Post-processing steps included filling holes to ensure object continuity, three successive erosions to refine boundaries, and a dilation step to restore shape integrity. The final processed mask was saved to a predefined directory, and the original and duplicated images were closed to optimize memory usage

Macro-4G (b) Generating DAPI Mask

To generate the DAPI mask, a single slice from the Z-stack containing the maximum number of nuclei. The following workflow was applied:

```
// Prompt user to select an image file
//open(getFile("Select an image file"));
// Get the title of the opened image (remove extension for consistency)
inputTitle = replace(getTitle(), ".tif", "");
// Duplicate the image, keeping only 1 channel
run("Duplicate...", "duplicate channels=1");
// Perform background subtraction
run("Subtract Background...", "rolling=100");
// Apply Gaussian blur
run("Gaussian Blur...", "sigma=1");
// Enhance local contrast using CLAHE
run("Enhance Local Contrast (CLAHE)", "blocksize=127 histogram=256 maximum=3 mask=*None* fast_(less_accurate)");
// Apply auto-thresholding using Otsu's method
setAutoThreshold("Otsu dark");
// Convert to mask
setOption("BlackBackground", false);
run("Convert to Mask");
// Define save path dynamically
savePath = "G:/Core/maya/Manuscript/003_Quantifications/Q42_FOXO-GFP_PD_AC2108/Step6-DAPI Mask/" + inputTitle + "-1.tif";
// Save the processed mask
saveAs("Tiff", savePath);
// Close the duplicated image
close();
// Close the original image
```

```
selectImage(inputTitle + ".tif");
close();
```

This script processes an image file by selecting a single channel, applying background subtraction (rolling ball radius = 100), and enhancing contrast using Gaussian blur ($\sigma = 1$) and CLAHE (Contrast Limited Adaptive Histogram Equalization). It then applies Otsu's auto-thresholding, converts the image to a binary mask, and saves the processed mask in a predefined directory. Finally, the script closes both the processed and original images to optimize memory usage.

Quantification Fig. 6F

Quantification of p-S6 Intensity in Engrailed and Non-Engrailed Pouch Regions:

1. Select slices with the maximum number of cells in the pouch of the disc.
2. Generate a mask for the engrailed region within the pouch.
3. Generate a mask for the non-engrailed pouch region.
4. Create tissue masks for both the engrailed and non-engrailed pouch regions using the DAPI channel.
5. Measure the p-S6 intensity using the engrailed and non-engrailed tissue masks.
6. Calculate the ratio of p-S6 intensity between the engrailed and non-engrailed pouch regions.

Same logic applied for quantification Fig 6D, 6K and 6L.

Macro-6F(a)- Generating tissue mask

```
run("Duplicate...", "duplicate channels=1");
run("Gaussian Blur...", "sigma=2");
setAutoThreshold("Percentile dark");
//setThreshold(36, 255);
setOption("BlackBackground", false);
run("Convert to Mask");
run("Dilate");
```

This macro duplicates the image, applies a Gaussian blur to reduce noise, and then automatically thresholds the image using the "Percentile dark" method. It converts the thresholded image into a binary mask and dilates the mask to expand the regions of interest. This prepares the image for further analysis by isolating and enhancing the relevant features.

Original Western Blots for Supp Fig S3.1 B

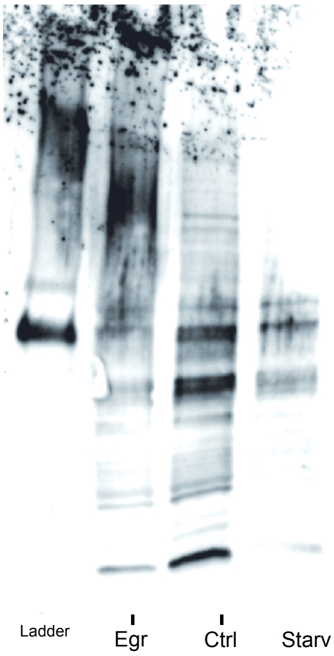

p-AKT

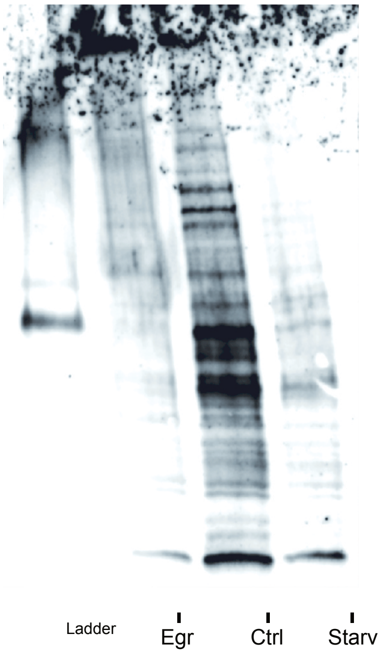

AKT
